# Bladder Agenesis: A Systematic Review

**DOI:** 10.7759/cureus.45121

**Published:** 2023-09-12

**Authors:** Majd H Yahya

**Affiliations:** 1 Pediatrics, Ibn Al Haytham Hospital, Amman, JOR

**Keywords:** ambiguous genitalia, urorectal septal malformation sequence, vitelline vascular steal, bladder agenesis, urogenital anomalies, congenital anomalies, vascular malformation, cloacal deformities

## Abstract

Bladder agenesis is a rare congenital deformity characterized by the absence of the bladder. It is primarily observed in postmortem dissections of stillbirths rather than live births. The condition is often associated with other congenital anomalies, leading to the hypothesis that most affected fetuses do not survive to term. However, the exact cause and specific associated anomalies remain unclear and poorly described in the literature. The limited mention of bladder agenesis in textbooks and literature underscores the importance of creating a comprehensive source for future research in this field. Therefore, our objective is to collect and analyze data on bladder agenesis, focusing on associated anomalies and potential causes, to enhance our understanding of the condition.

We conducted a thorough review of reports collected from three databases, Google Scholar, PubMed, and Science Direct, last searched on July 30, 2023, starting with 327 reports. Excluding duplicates and records written in languages other than English, veterinary studies, irrelevant reports, or stillbirths. Inclusion criteria were the following: cases must have proven bladder agenesis, not hypoplasia, and must have most of the information, including the age of diagnosis, presenting symptoms, gender, associated anomalies, and management or outcome of the patient. A quality assessment was conducted according to the Joanna Briggs Institute checklist for case reports. A total of 65 case reports from 56 articles were included in the review.

Through our manual analysis, we documented a wild array of malformations associated with bladder agenesis. Among the reports reviewed, 93% exhibited urinary system malformations beside bladder agenesis, 77% were found to have reproductive malformations, 44% had gastrointestinal anomalies, 38% showed musculoskeletal malformations, 28% had cardiac malformations, and another 28% had vascular anomalies. The overall mortality rate was 38%, with a higher rate of 74% for males compared to 20% for females. By collating and analyzing those case reports, we aim to contribute to a better understanding of bladder agenesis and its associated anomalies, facilitating further investigations and advancements in the field.

## Introduction and background

Throughout the years, there have been few reported cases of bladder agenesis, with the earliest mention dating back to 1654 by Rhodius. It is difficult to pinpoint an accurate number of cases that have been reported since then. There have been few citations of live births in English literature, for which it has been considered an extremely rare condition, especially given that it has been associated with numerous anomalies, some of which are fatal.

## Review

Methodology

**Figure 1:**
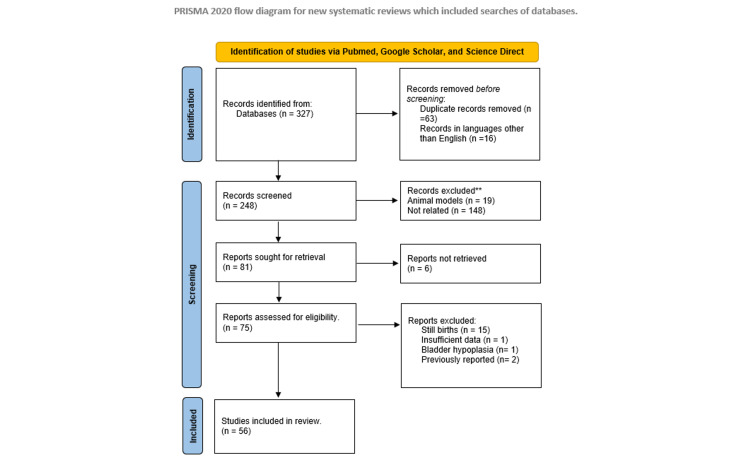
Flowchart of database search results for reports of bladder agenesis according to the PRISMA guidelines From: Page MJ, McKenzie JE, Bossuyt PM, Boutron I, Hoffmann TC, Mulrow CD, et al. The PRISMA 2020 statement: an updated guideline for reporting systematic reviews. BMJ 2021;372:n71. doi: 10.1136/bmj.n71. For more information, visit http://www.prisma-statement.org/. PRISMA: Preferred Reporting Items for Systematic Reviews and Meta-Analyses.

This systematic review was conducted in accordance with the Preferred Reporting Items for Systematic Reviews and Meta-Analyses (PRISMA) 2020 guidelines (Figure 1 and Tables 1, 2). The search last conducted on July 30, 2023, encompassed three prominent databases: Google Scholar, PubMed, and ScienceDirect. Our query focused on case reports, utilizing precise keywords, including "bladder agenesis," "-gall," and "-gallbladder." A comprehensive screening process was undertaken by a single reviewer to eliminate duplicate entries, publications in languages other than English, studies involving animal models, and articles that were irrelevant or inaccessible in full text. Furthermore, we exercised prudence in excluding articles related to stillbirths, intrauterine scans, urinary bladder hypoplasia, and those presenting insufficient data for analysis. In addition, articles that reported the same case were analyzed for further details without duplication within the statistical analysis. To include a study, it must specify that it was indeed a case of bladder agenesis and include most, if not all, of the following information: age of diagnosis, presenting symptom, gender, associated anomalies, and management or outcome of the patient. Additionally, a quality assessment of the reports was conducted according to the Joanna Briggs Institute (JBI) critical appraisal tools for systematic reviews, using their checklist for case reports (Table 3). The data were manually collected and sorted in a tabular manner in Excel (Microsoft Corporation, Redmond, WA) for easier data extraction and comparison.

**Table 1:** PRISMA 2020 main checklist From: Page MJ, McKenzie JE, Bossuyt PM, Boutron I, Hoffmann TC, Mulrow CD, et al. The PRISMA 2020 statement: an updated guideline for reporting systematic reviews. MetaArXiv. 2020, September 14. DOI: 10.31222/osf.io/v7gm2. For more information, visit www.prisma-statement.org. PRISMA: Preferred Reporting Items for Systematic Reviews and Meta-Analyses.

Topic	No.	Item	Location where the item is reported
TITLE			
Title	1	Identify the report as a systematic review.	Title
ABSTRACT			
Abstract	2	See the PRISMA 2020 for the abstract checklist.	Table No. 2
INTRODUCTION			
Rationale	3	Describe the rationale for the review in the context of existing knowledge.	Abstract paragraph 1
Objectives	4	Provide an explicit statement of the objective(s) or question(s) the review addresses.	Abstract paragraph 1
METHODS			
Eligibility criteria	5	Specify the inclusion and exclusion criteria for the review and how studies were grouped for the syntheses.	Methodology
Information sources	6	Specify all databases, registers, websites, organizations, reference lists, and other sources searched or consulted to identify studies. Specify the date when each source was last searched or consulted.	Methodology
Search strategy	7	Present the full search strategies for all databases, registers, and websites, including any filters and limits used.	Methodology
Selection process	8	Specify the methods used to decide whether a study met the inclusion criteria of the review, including how many reviewers screened each record and each report retrieved, whether they worked independently, and if applicable, details of automation tools used in the process.	Methodology
Data collection process	9	Specify the methods used to collect data from reports, including how many reviewers collected data from each report, whether they worked independently, any processes for obtaining or confirming data from study investigators, and if applicable, details of automation tools used in the process.	Methodology
Data items	10a	List and define all outcomes for which data were sought. Specify whether all results that were compatible with each outcome domain in each study were sought (e.g. for all measures, time points, and analyses), and if not, the methods used to decide which results to collect.	Table No. 3
	10b	List and define all other variables for which data were sought (e.g. participant and intervention characteristics and funding sources). Describe any assumptions made about any missing or unclear information.	Methodology
Study risk of bias assessment	11	Specify the methods used to assess the risk of bias in the included studies, including details of the tool(s) used, how many reviewers assessed each study and whether they worked independently, and if applicable, details of automation tools used in the process.	Methodology
Effect measures	12	Specify for each outcome the effect measure(s) (e.g. risk ratio and mean difference) used in the synthesis or presentation of results.	N/A
Synthesis methods	13a	Describe the processes used to decide which studies were eligible for each synthesis (e.g. tabulating the study intervention characteristics and comparing against the planned groups for each synthesis (item 5)).	N/A
	13b	Describe any methods required to prepare the data for presentation or synthesis, such as handling of missing summary statistics, or data conversions.	Methodology
	13c	Describe any methods used to tabulate or visually display the results of individual studies and syntheses.	Methodology
	13d	Describe any methods used to synthesize results and provide a rationale for the choice(s). If meta-analysis was performed, describe the model(s), method(s) to identify the presence and extent of statistical heterogeneity, and software package(s) used.	Methodology
	13e	Describe any methods used to explore possible causes of heterogeneity among study results (e.g. subgroup analysis and meta-regression).	N/A
	13f	Describe any sensitivity analyses conducted to assess the robustness of the synthesized results.	N/A
Reporting bias assessment	14	Describe any methods used to assess the risk of bias due to missing results in a synthesis (arising from reporting biases).	Methodology
Certainty assessment	15	Describe any methods used to assess certainty (or confidence) in the body of evidence for an outcome.	N/A
RESULTS			
Study selection	16a	Describe the results of the search and selection process, from the number of records identified in the search to the number of studies included in the review, ideally using a flow diagram.	Figure No. 1
	16b	Cite studies that might appear to meet the inclusion criteria, but which were excluded, and explain why they were excluded.	N/A
Study characteristics	17	Cite each included study and present its characteristics.	Table No. 5
Risk of bias in studies	18	Present assessments of risk of bias for each included study.	Table No. 3
Results of individual studies	19	For all outcomes, present, for each study: (a) summary statistics for each group (where appropriate) and (b) an effect estimate and its precision (e.g. confidence/credible interval), ideally using structured tables or plots.	N/A
Results of syntheses	20a	For each synthesis, briefly summarize the characteristics and risk of bias among contributing studies.	N/A
	20b	Present results of all statistical syntheses conducted. If meta-analysis was done, present for each the summary estimate and its precision (e.g. confidence/credible interval) and measures of statistical heterogeneity. If comparing groups, describe the direction of the effect.	Conclusion paragraphs 1 and 2
	20c	Present results of all investigations of possible causes of heterogeneity among study results.	N/A
	20d	Present results of all sensitivity analyses conducted to assess the robustness of the synthesized results.	N/A
Reporting biases	21	Present assessments of risk of bias due to missing results (arising from reporting biases) for each synthesis assessed.	N/A
Certainty of evidence	22	Present assessments of certainty (or confidence) in the body of evidence for each outcome assessed.	N/A
DISCUSSION			
Discussion	23a	Provide a general interpretation of the results in the context of other evidence.	N/A
	23b	Discuss any limitations of the evidence included in the review.	Limitation paragraph 1
	23c	Discuss any limitations of the review processes used.	Limitation paragraph 1
	23d	Discuss the implications of the results for practice, policy, and future research.	Limitation paragraphs 2 and 3
OTHER INFORMATION			
Registration and protocol	24a	Provide registration information for the review, including the register name and registration number, or state that the review was not registered.	N/A
	24b	Indicate where the review protocol can be accessed, or state that a protocol was not prepared.	N/A
	24c	Describe and explain any amendments to information provided at registration or in the protocol.	N/A
Support	25	Describe sources of financial or non-financial support for the review, and the role of the funders or sponsors in the review.	Disclosures
Competing interests	26	Declare any competing interests of review authors.	Disclosures
Availability of data, code, and other materials	27	Report which of the following are publicly available and where they can be found: template data collection forms; data extracted from included studies; data used for all analyses; analytic code; any other materials used in the review.	N/A

**Table 2:** PRISMA abstract checklist From: Page MJ, McKenzie JE, Bossuyt PM, Boutron I, Hoffmann TC, Mulrow CD, et al. The PRISMA 2020 statement: an updated guideline for reporting systematic reviews. MetaArXiv. 2020, September 14. DOI: 10.31222/osf.io/v7gm2. For more information, visit www.prisma-statement.org. PRISMA: Preferred Reporting Items for Systematic Reviews and Meta-Analyses.

Topic	No.	Item	Reported?
TITLE			
Title	1	Identify the report as a systematic review.	Yes
BACKGROUND			
Objectives	2	Provide an explicit statement of the main objective(s) or question(s) the review addresses.	Yes
METHODS			
Eligibility criteria	3	Specify the inclusion and exclusion criteria for the review.	Yes
Information sources	4	Specify the information sources (e.g. databases and registers) used to identify studies and the date when each was last searched.	Yes
Risk of bias	5	Specify the methods used to assess the risk of bias in the included studies.	Yes
Synthesis of results	6	Specify the methods used to present and synthesize results.	Yes
RESULTS			
Included studies	7	Give the total number of included studies and participants and summarize relevant characteristics of studies.	Yes
Synthesis of results	8	Present results for main outcomes, preferably indicating the number of included studies and participants for each. If meta-analysis was done, report the summary estimate and confidence/credible interval. If comparing groups, indicate the direction of the effect (i.e. which group is favored).	Yes
DISCUSSION			
Limitations of evidence	9	Provide a brief summary of the limitations of the evidence included in the review (e.g. study risk of bias, inconsistency, and imprecision).	Yes
Interpretation	10	Provide a general interpretation of the results and important implications.	Yes
OTHER			
Funding	11	Specify the primary source of funding for the review.	Yes
Registration	12	Provide the register name and registration number.	No

**Table 3:** Quality assessment of the reports included according to the Joanna Briggs Institute (JBI) checklist of case reports

Author	Reference number	Demographic description	History timeline	Condition at presentation	Diagnostic tests description	Interventions description	Post-intervention condition/outcome	Provides takeaway lessons	Overall appraisal
Miller	[1]	Yes	Yes	Yes	Yes	Yes	Yes	No	Included
Glenn	[2]	Yes	Yes	Yes	Yes	Yes	Yes	Yes	Included
Palmer	[3]	Yes	Yes	Yes	Yes	Yes	Yes	Yes	Included
Graham	[4]	Yes	Yes	Yes	Yes	Yes	Yes	Yes	Included
Vakili	[5]	Yes	No	Yes	Yes	Yes	Yes	Yes	Included
Tortora	[6]	Yes	No	Yes	Yes	Yes	Yes	Yes	Included
Metoki	[7]	Yes	Yes	Yes	Yes	No	Yes	Yes	Included
Dusmet	[8]	Yes	Yes	Yes	Yes	No	Yes	Yes	Included
Aragona	[9]	Yes	Yes	Yes	Yes	No	Yes	Yes	Included
Krull	[10]	Yes	Yes	Yes	Yes	No	Yes	Yes	Included
Akdas	[11]	Yes	Yes	Yes	Yes	Yes	No	Yes	Included
Dykes	[12]	Yes	No	Yes	Yes	No	No	Yes	Included
Gopal	[13]	Yes	Yes	Yes	Yes	Yes	Yes	Yes	Included
Cilento	[14]	Yes	No	Yes	Yes	Yes	Yes	Yes	Included
Sarica	[15]	Yes	Yes	Yes	Yes	No	No	Yes	Included
Bhagwat	[16]	Yes	Yes	Yes	Yes	No	Yes	Yes	Included
Kaefer	[17]	Yes	Yes	Yes	Yes	Yes	Yes	Yes	Included
Paşaoglu	[18]	Yes	Yes	Yes	Yes	No	No	Yes	Included
Kasat	[19]	Yes	Yes	Yes	Yes	Yes	Yes	Yes	Included
Karaguzel	[20]	Yes	Yes	Yes	Yes	Yes	Yes	Yes	Included
Benedetto	[21]	Yes	No	Yes	Yes	No	Yes	Yes	Included
Rennert	[22]	Yes	Yes	Yes	Yes	No	Yes	Yes	Included
Savanelli	[23]	Yes	Yes	Yes	Yes	Yes	Yes	Yes	Included
Nazif	[24]	Yes	Yes	Yes	Yes	Yes	Yes	Yes	Included
Rodin	[25]	Yes	Yes	Yes	Yes	Yes	Yes	Yes	Included
Weight	[26]	Yes	Yes	Yes	Yes	Yes	Yes	Yes	Included
Khemchandani	[27]	Yes	Yes	Yes	Yes	Yes	Yes	Yes	Included
Patkowski	[28]	Yes	Yes	Yes	Yes	Yes	Yes	Yes	Included
Jain	[29]	Yes	Yes	Yes	Yes	No	Yes	Yes	Included
Rezaie	[30]	Yes	No	Yes	Yes	No	No	Yes	Included
Barber	[31]	Yes	Yes	Yes	Yes	Yes	Yes	Yes	Included
Chen	[32]	Yes	Yes	Yes	Yes	Yes	Yes	Yes	Included
Nazim	[33]	Yes	Yes	Yes	Yes	Yes	Yes	Yes	Included
Pfister	[34]	Yes	Yes	No	Yes	Yes	Yes	Yes	Included
Indiran	[35]	Yes	No	Yes	Yes	Yes	No	Yes	Included
García-de León Gómez	[36]	Yes	Yes	Yes	Yes	Yes	Yes	Yes	Included
Baheti	[37]	Yes	Yes	Yes	Yes	Yes	Yes	Yes	Included
Sandal	[38]	Yes	Yes	Yes	Yes	Yes	Yes	Yes	Included
Pandey	[39]	Yes	Yes	Yes	Yes	Yes	Yes	Yes	Included
Priyadarshi	[40]	Yes	Yes	Yes	Yes	Yes	Yes	Yes	Included
Crocoli	[41]	Yes	Yes	Yes	Yes	Yes	Yes	Yes	Included
Pacheco-Mendoza	[42]	Yes	Yes	Yes	Yes	Yes	Yes	Yes	Included
Ghasi	[43]	Yes	Yes	Yes	Yes	No	No	Yes	Included
Sailo	[44]	Yes	No	Yes	Yes	No	No	Yes	Included
Khan	[45]	Yes	Yes	Yes	Yes	Yes	Yes	Yes	Included
Friedman	[46]	Yes	Yes	Yes	Yes	Yes	Yes	Yes	Included
Singh	[47]	Yes	Yes	Yes	Yes	Yes	Yes	Yes	Included
Yurtcu	[48]	Yes	Yes	Yes	Yes	Yes	Yes	Yes	Included
Atıcı	[49]	Yes	Yes	Yes	Yes	Yes	Yes	Yes	Included
Lowrey	[50]	Yes	Yes	Yes	Yes	Yes	Yes	Yes	Included
Nazer	[51]	Yes	Yes	Yes	Yes	Yes	Yes	Yes	Included
Gowtham	[52]	Yes	Yes	Yes	Yes	Yes	Yes	Yes	Included
Delshad	[53]	Yes	Yes	Yes	Yes	Yes	Yes	Yes	Included
Ozcakir	[54]	Yes	Yes	Yes	Yes	Yes	Yes	Yes	Included
Omil-Lima	[55]	Yes	Yes	Yes	Yes	Yes	Yes	Yes	Included
Ramya	[56]	Yes	Yes	Yes	Yes	Yes	Yes	Yes	Included

Results

Epidemiology

Most reports were from India with 14 cases, followed by the USA with 13 cases, Türkiye with eight, Italy with three, and Iran and Mexico with two reports each. One report was from each of the following countries: Germany, France, Poland, South Africa, Japan, Taiwan, Pakistan, KSA, UK, Switzerland, and Canada. The origin could not be determined in three cases.

It has been reported in some of the previous literature that the female-to-male ratio is 30:1. The reviewed reports had 19 males out of the 65 (two were of undermined gender), making the ratio 2.3:1. This female predominance has been attributed to the higher severity of associated anomalies in males, leading to intrauterine dismissal. Only seven subjects had a twin, five of whom were monozygotic [14,15,21,34,39], and one via in vitro fertilization [32]. One has hypospadias [21], one, similarly to their twin, has teratology of Fallot [14], and five were without complaint [15,29,32,37,45].

The mean age of presentation was approximately 4.5 years, with a median of 10 days and a mode of 0, with 32 subjects presenting at birth. The oldest subject was 60 years old at the time of the presentation.

Classifications

Metoki et al. [7] classified bladder agenesis according to the site of ureter insertion into five types in females: (1) remnant cloacal type: persistent cloaca due to failure of the urorectal septum; hence, the ureters open into the remnant cloaca; (2) rectal type; (3) urogenital type: the ureter opening into the urethra or the vaginal vestibulum; (4) vaginal type; and (5) ectopic type: ureters open into the uterus.

With 44 females, 21 were reported to have the vagina as the insertion site [1,2,5,6,11,24,27,30,32-35,37,41-43,51-53], accounting for 48% of the female cases; the urogenital sinus in nine cases or 21% of female cases [6,9,12,15,23,40,44,46], three cases reported the common cloaca as the site of insertion by Jain et al. [29] and ectopically to the skin at the natal cleft [16] or uterus [19]. Karaguzel et al. reported a case in which the subject had a blind pouch between the natal cleft and the coccyx, presumed to be of the anus, from which some urine was draining [20]. One case had bilateral renal and ureteric agenesis [29]. The remainder of the cases did not mention the site of insertion (Table 4).

**Table 4:** Summary of the reports included in the review with the main anomalies, gender, and age at the time of the report * The subject was assigned to be female according to the external genitalia; for the purposes of the statistical analysis, the biological gender was used. F: female; M: male; d: days; w: weeks; m: months; y: years; Lt: left; Rt: right; VSD: ventricular septal defect; PDA: patent ductus arteriosus; ASD: atrial septal defect; TOF: tetralogy of Fallot; VACTERL: vertebral, anal atresia, cardiac, tracheoesophageal, renal, and limbs deformities; PUJ: pelviureteric junction; DDH: developmental dysplasia of the hip; VATER: vertebral, anal, tracheoesophageal, radial, and renal anomalies.

Case number	Author	Article number	Gender	Age of presentation	Vascular	Urinary malformations	Site of insertion	Genital malformations	Anal anomalies	Vertebral malformations	Limb malformations	Cardiac malformations	Other malformations	Reported syndrome or sequence
1	Miller	[1]	F	27 y		Hydroureteronephrosis, renal abscess	1 anterior vaginal wall	Small cervix	Poor tone	Spinal bifida, scoliosis	Bilateral equinovarus deformities			
2	Glenn	[2]	F	3.5 y		Hydroureteronephrosis, duplication of Lt upper collecting system, blind-ended urethra.	4 openings in the vagina, 2 of which are fistulas to other openings	Bicornuate uterus				Small VSD	Meckel's diverticulum	
3	Palmer	[3]	F	23 y				Bicornuate uterus	Absent posterior fourchette	Scoliosis, additional vertebrae	Lt little finger 1 cm shorter		Café' au lait spots	
4	Graham	[4]	F	6 w		Solitary kidney with hydronephrosis, and normal urethral opening	Urethra							
5	Vakili	[5]	F	10 y	Absent Lt common iliac artery; internal and external iliac arteries originated from the aorta. The external iliac has an unusual medial course	Ureters join in the midline and open into the vagina, absent urethra	Anterior vaginal wall, crescent in shape	Blind-ended vagina, cervix absent, uterus small, ovaries normal						
6	Tortora	[6]	F	1 m		Bilateral renal dysplasia	Vaginal horns	Prominent mons pubis, protruding skin fold, duplicate vagina and uterus					Malrotation with colonic duplication	
7	Tortora	[6]	F	Newborn		Renal dysplasia with tortuous ureters	Urogenital sinus	Absent labia majora, hypoplastic labia minora, hyperplastic skin fold in the area of the clitoris, and stenotic cervix						
8	Tortora	[6]	F	1 y		Hydroureteronephrosis	Urogenital sinus					Congenital heart disease (unspecified)	Cleft palate and strabismus	
9	Metoki	[7]	F	4 m		Solitary kidney with hydroureteronephrosis		Protruding skin fold, a single ovary, and a normal uterus. Single urogenital opening	Atresia					
10	Dusmet	[8]	M 46 XY	Newborn	Single umbilical artery	Bilateral renal dysplasia with blind ureter		Absent phallus, rudimentary, and intra-abdominal testes	Atresia	Scoliosis, supernumerary hemivertebra, partial fusion of ribs, several vertebrae, and of the sacrum	Lobster claw	PDA	Tracheoesophageal fistula with partial proximal esophageal atresia; bilateral cleft lip with cleft palate; broad, flat nose; bilateral epicanthic folds; dysplastic, low-set ears; redundant loose skin at the back of the neck	VATER
11	Aragona	[9]	F	Newborn		Solitary cystic dysplastic kidney with tortuous ureter	Urogenital sinus	Hypertrophied clitoris and bicornuate uterus						
12	Krull	[10]	F 46 XX	12 d		Bilateral hydroureteronephrosis, absent urethra		Blind-ended vagina, cervix absent, uterus and ovaries hypoplastic					Hirschsprung's	
13	Akdas	[11]	F	30 y	Absent Rt renal artery	Solitary small kidney, absent urethra	The left fornix of the vagina	Bilateral polycystic ovaries						
14	Dykes	[12]	F		Aberrant left iliac artery arising from the aberrant aorta low in the pelvis. Lt internal iliac branch absent	Fused renal ectopia	Urogenital sinus	Bicornuate uterus, right side absent, ovaries and fallopian tubes present bilaterally						
15	Gopal	[13]	M	2.5 y		Bilateral hydroureteronephrosis, Lt dysplastic ectopic pelvic, distended posterior urethra	The distended posterior urethra	Hypospadias, seminal vesicles distended bilaterally with a cyst. The ejaculatory ducts are inserted into ureters						
16	Cilento	[14]	M	Newborn	Single umbilical artery	Solitary dysplastic kidney, urethra patent		Penoscrotal transposition		Additional rib and a sacral dimple	Absent Lt thumb	Teratology of Fallot	Esophageal atresia and redundant rectum	
17	Sarica	[15]	F	12 y		Solitary ectopic hydronephrotic kidney. The urethra blind ended	Urethra			Lt hemihypertrophy, scoliosis, spinal bifida, and first lumber vertebrae rib	Absent Lt thumb	Small membranous VSD	Ear tags and grade II goiter	
18	Bhagwat	[16]	F 46 XX	Newborn		Solitary ectopic kidney, urethra absent	Skin over the natal cleft	Vaginal atresia	Anorectal malformation	Spina bifida			Colonic duplication	
19	Kaefer	[17]	M 46 XY	Newborn	Duplicated aorta and internal iliac arteries join as a common vessel	Unilateral hydroureteronephrosis, the other side; cystic dysplastic. The urethra is absent	A fibrous cord into the anterior wall of the rectum	Penoscrotal transposition, small skin tag anterior to the rectum, descended testis with a paucity of spermatogonia and Leydig cells						
20	Paşaoglu	[18]	M	60 y		Unilateral hydroureteronephrosis, the other side; dysplastic	Prostatic urethra							
21	Kasat	[19]	F	20 d		Bilateral hydroureteronephrosis, Rt enclosed by urinoma	Rt into the uterus, Lt ends with stenosis	Distal vaginal atresia, proximal part, and uterus filled with urine						
22	Karaguzel	[20]	F 46 XX	Newborn		Bilateral renal ectopia with increased echogenicity, one is dysplastic	Undetermined	Absence of labia minora and majora and protruding skin fold in the area of the clitoris, lack of perineal openings. Absent vagina and uterus. Ovaries are normal, fallopian tube connected like a cord	Rectal atresia	Additional rib, vertebrae fusion	Foot agenesis, and extremity remnant	Small VSD		
23	Benedetto	[21]	M 46 XY	Newborn		Bilateral renal agenesis		Absent phallus with normal scrotum and testes	Anorectal agenesis without fistula					
24	Rennert	[22]	M 46 XY	Newborn		Bilateral multicystic dysplastic, third kidney also cystic dysplastic	Rectum	Absent phallus with normal scrotum and testes	Posterior displacement	Additional rib	Proximal insertion of the thumbs and subluxation of wrists and elbows		Dolichocephalous with prominent occiput, epicanthic folds, posterior angulation of the ears, and a short neck	Treacher Collins
25	Savanelli	[23]	F	4 w		Renal ectopia with tortuous ureters, "vanishing Lt kidney"	Urogenital sinus	Prominent clitoris, a single perineal opening, and a bicornuate uterus	Anterior anus	Vertebral hemispondylosis and sacral dysmorphism				
26	Nazif	[24]	F	Newborn		Solitary cystic dysplastic kidney with tortuous ureter. The urethra is absent	Vestibule	Blind-ended vagina. Uterus and cervix absent. Fallopian tubes blind-ended, ovaries cystic						
27	Rodin	[25]	M 46 XY	Newborn	Anomalous aorta below the inferior mesenteric artery, coursed anteriorly throughout the abdomen and pelvis, veered to the left pelvis, and bifurcated into the common iliac	Echogenic solitary duplex left kidney	Terminate in the sacral area	Penoscrotal transposition with suprapubic maldeveloped scrotum and ectopically located phallus in the sacral area				Large PDA		
28	Weight	[26]	M 46 XY	Newborn		Solitary dysplastic kidney with focal cystic dilations of the ureter	Anterior rectal wall		Anal stenosis				Bilateral inguinal hernia	
29	Khemchandani	[27]	F	2 y		Solitary hydroureteronephrosis kidney	Vestibule							
30	Patkowski	[28]	M 46 XY	Newborn		Bilateral cystic dysplasia kidneys with urethral stenosis	Rectum	Penoscrotal transposition with bifid scrotum and a thick vas deferens						
31	Jain	[29]	M	Newborn		Bilateral hypoplastic dysplastic kidneys	Common cloaca	Absent phallus with cryptorchidism	Rectal atresia		Contractures in both hands and feet		Ileal stricture, Meckel’s diverticulum, dysmorphic facies with bilateral upward slant of eyes, depressed nasal bridge, and low-set ears	Urorectal septal malformation
32	Jain	[29]	F	Newborn			Common cloaca	Absent perineal opening			Bilateral cubitus valgus, clinodactyly, and rocker bottom feet	ASD, atrioventricular canal defect of ostium primum type, and tricuspid stenosis	Pouch colon, low-set ears, webbed neck	Urorectal septal malformation
33	Jain	[29]	F	Newborn		Solitary cystic dysplastic kidney with ureteric atresia and PUJ stenosis	Common cloaca	Phallus-like structure, fused labia, a single midline perineal opening, rudimentary uterus, and ovaries	Stenotic rectal opening to cloacal sac		Accessory thumb		Low-set ears	Urorectal septal malformation
34	Jain	[29]	F	Newborn	Single umbilical artery	Solitary dysplastic kidney. The urethra is absent	Common cloaca	Vaginal agenesis, and bilateral streak ovaries	Imperforated anus	Spinal bifida and meningomyelocele	Bilateral equinovarus deformities			Urorectal septal malformation
35	Jain	[29]	F	Newborn	Single umbilical artery	Bilateral renal agenesis		Clitoral hypertrophy, no perineal opening with bifid, atretic vagina	Imperforated anus		Hypoplastic right thumb; constriction at the base of the left thumb, absent phalanges, bilateral dislocation of the hips, and genu valgum		Low-set ears, esophageal atresia with tracheoesophageal fistula type 3, and adrenals were bean-shaped and occupied the entire renal bed. Small intestine showed stenoses and dilatations at multiple sites	Urorectal septal malformation
36	Rezaie	[30]	F	1.5 y		Solitary hyperplastic hypo nephrotic kidney with tortuous ureter. The urethra is absent	Vagina	Stenotic orifice at the vaginal vestibule						
37	Rezaie	[30]	F	6 y		Unilateral mild hydronephrosis with unilateral duplicate ureter. The urethra is absent	2 ureteral opening at the vaginal vestibule							
38	Barber	[31]	M	Newborn		Unilateral cystic, bilateral dysplastic kidneys	Prostatic urethra					VSD		
39	Chen	[32]	F	1 m		Unilateral hypoplastic, bilateral hydronephrosis with tortuous ureters	Vaginal vestibule							
40	Nazim	[33]	F 46 XX	8 y		Bilateral hydronephrosis, one kidney is non-functional	Anterior vaginal wall	Urine reflux into fallopian tubes						
41	Pfister	[34]	F	3 m			Anterior vaginal wall			Scoliosis, malformations of ribs and vertebrae, and suspected DDH				
42	Indiran	[35]	F	12 y	High aortic bifurcation, anomalous right internal iliac artery, Lt kidney supplied by accessory artery in addition to renal artery	Solitary kidney with calyceal dilatation	Anterior vaginal wall	Unicornuate		DDH with gluteal and thigh muscle atrophy				
43	García-de León Gómez	[36]	M	Newborn		Kidneys with increased echogenicity, multi-simple cystic formation, and Lt ectopic. Perineal urinary meatus		Absent phallus and scrotal raphe	Imperforated anus			Patent foramen ovale and PDA		
44	Baheti	[37]	F	3 y		Hydroureter	Vagina	Blind-ended vagina and absent uterus						
45	Sandal	[38]	M 46 XY	Newborn	Single umbilical artery	Bilateral renal agenesis and absent urethra		Distended penis with absent scrotum		Butterfly vertebras	Unilateral preaxial polydactyly, and bilateral equinovarus deformities	ASD		VACTERL
46	Pandey	[39]	M 46 XY	Newborn	Single umbilical artery	Bilateral renal agenesis		Absent external genitalia	Imperforated anus	Partial sacrococcygeal agenesis	Single lower limb, short tibia showing distal shaft tapering, and absent fibula, ankle, and foot bones			Sirenomelia
47	Priyadarshi	[40]	F 46 XX	5 y		Bilateral hydroureteronephrosis and absent urethra	Urogenital sinus							
48	Crocoli	[41]	F 46 XX	Newborn		Hydronephrosis, small bilateral subcortical renal cysts, and a unilateral tortuous ureter	Vagina	Clitoral hypertrophy and no perineal opening	Posterior displacement				Esophageal atresia (dilated upper esophageal pouch)	
49	Pacheco-Mendoza	[42]	F	12 y		Bilateral incomplete renal duplication and unilateral ectopic kidney. The urethra is blind ended	Anterior vaginal wall							
50	Ghasi	[43]	F	22 y		Bilateral hydronephrosis with unilateral calcified ureter and pelvicalyceal system, the urethra is normally positioned	Vagina	Dilated vagina						
51	Sailo	[44]	F	9 m		Ectopic renal lump with absent urethra	Urogenital sinus			Butterfly and block vertebrae				
52	Khan	[45]	M	Newborn		Cystic dysplastic horseshoe pelvic kidney without corticomedullary differentiation, and bilateral ectopic ureters with absent urethra		Small phallus with undescended testes		Butterfly and hemi vertebrae		VSD with tricuspid valve insufficiency	Up-slanting palpebral fissures, a long philtrum, and a thin upper lip	
53	Friedman	[46]	F 46 XX	Newborn	Esophageal ring; left aortic arch and aberrant right subclavian artery coursing posterior to the esophagus	Unilateral cystic, bilateral dysplastic kidneys with solitary refluxing ureter	Stenotic urogenital sinus 1.5 cm in length	Fused labia majora with a skin tag posteriorly, absent clitoris (transposition of external genitalia)	Displaced anteriorly					
54	Singh	[47]	M	Newborn		Increased renal echogenicity with multiple simple cysts and absent urethra		Absent phallus, normal scrotum, and descended testes	Anus opening absent, gluteal cleft, and anal dimple not well developed					Urorectal septal malformation
55	Yurtcu	[48]	F	Newborn		Unilateral PUJ obstruction			Imperforated anus					Urorectal septal malformation
56	Atıcı	[49]	Undetermined	Newborn	Single umbilical artery	Bilateral renal agenesis		Absent external genitalia	Single genital patency just below the coccyx, connected to the colon		Two femurs, two tibia, one fibula, one foot, and four toes	Aortic coarctation		Sirenomelia
57	Lowrey	[50]	F	8 y	Absent Lt common iliac, artery from Rt internal iliac to Lt external iliac	Unilateral cystic dysplastic kidney, the other is ectopic						TOF and pulmonary stenosis		
58	Lowrey	[50]	F	6 y	Pulsatile artery traversing where the bladder should have been	Bilateral dysplasia kidneys		Vaginal agenesis	Imperforated anus					
59	Nazer	[51]	F 46 XX	1 m	Low bifurcation in the left iliac fossa with absent internal iliac arteries bilaterally	Unilateral dysplastic kidney with short urethra	Anterosuperior vaginal wall	Distal vaginal atresia with uterus didelphys unilateral hematocolpos	Low imperforated anus and stenosis					
60	Gowtham	[52]	F	20 y		Solitary kidney with distally dilated ureter	Vestibule	Stenotic vaginal opening with labial adhesions		DDH				
61	Delshad	[53]	F	3 y			Vagina		Imperforated anus	Sacral hypoplasia				
62	Delshad	[53]	F	6 y			Vagina							
63	Ozcakir	[54]	Undetermined	Newborn		Bilateral renal agenesis with absent urethra		Smooth perineal, a small nubbin of tissue	Absent perineal orifices				Unilateral choanal atresia	Urorectal septal malformation
64	Omil-Lima	[55]	M	Newborn		Bilateral cystic dysplastic kidneys, the urethral meatus was orthotopic	Dilated seminal vesicle bilaterally	Penoscrotal transposition with scrotum bifid and descended testes bilaterally	Imperforated anus with perineal fistula					
65	Ramya	[56]	M	Newborn	Single umbilical artery	Bilateral cystic dysplastic kidneys	Common cloaca	Absent phallus with well-developed scrotum, testes one descended, the other is atrophic	Imperforated anus	Sacral agenesis	Unilateral absent fibula (hemimelia), and bilateral elbow contractures			Urorectal septal malformation

There are three types in males: (1) remnant cloacal type; (2) rectal type; and (3) urogenital sinus type.

Of the 19 male subjects, five were not investigated for the sites of insertion [14,25,36,45,47]; three were reported with the rectum as the site of insertion [22,26,28]; and another case had no apparent outlet but had a fibrous cord connecting to the rectum [17]. Three subjects had ureters attached to the urethra [13,18,31] and one to the seminal vesicle [54]. Those four latter cases represent the urogenital sinus type. Two cases reported the common cloaca as the site of insertion [29,56]. In one case, the ureters were blind-ended [8]. Three cases reported bilateral renal agenesis (Table 4) [21,38,39].

Subjects could be divided into two groups depending on the timing of the presentation: (1) early presentation as neonates due to one or more of the following: azotemia or oligohydramnios and its complications [6,9,16,19,22,24-26,28,29,31,39,45-47,54-56] or ambiguous genitalia [6,8,9,14,17,20,21,25,28,29,36,38,39,41,49,51,54] and occasionally part of a complex syndrome; in those cases, absent bladder was an incidental finding, or less commonly, subjects who were noted to have imperforated anus or inability to pass stool [9,20,28,36,48,51]. The prenatal presentation was due to oligohydramnios, and the definitive diagnosis was after birth. (2) Late presentation: beyond the first year of life, usually with incontinence [2,3,5,11,15,18,37,40,52,53], urinary tract infection (UTI) [1,6,30,34,43], or both [13,27,30,33,35,42].

The period of infancy is a mixture. Graham had a subject at six weeks of age with failure to gain weight with both UTI and azotemia in addition to high blood pressure [4]. Similarly, in another study by Savanelli et al., the subject presented at four weeks with ambiguous genitalia, azotemia, and UTI [23]. Cases of UTI were noted as early as the first month [10,32]. One subject had recurrent UTIs at the age of three months [34]. Metoki et al.’s subject presented at four months with urogenital abnormalities, azotemia, and metabolic acidosis [7], and a case of continuous dribbling at nine months by Sailo and Sailo [44].

Associated Anomalies

Bladder agenesis has been found to be associated with a wide array of malformations, including those of the urogenital system, cardiovascular system, musculoskeletal system, and gastrointestinal system (Table 4).

Gastrointestinal Anomalies

There are 20 reports of anorectal malformation as the imperforated anus or anal atresia [7,8,16,20,21,29,31,36,39,47,48,50,53-56], anal stenosis [26], and an anteriorly located anus was described in two instances [23,46] and posteriorly once [22]. Rectal abnormalities were redundant [14], stenotic and opening to cloaca [29], posteriorly displaced [41], and agenesis [29]. Hirschsprung has been reported in one case by Krull et al. to be associated with bladder agenesis [10]. Colonic pouch [29] and duplication have been reported as well [6,16]. Intestinal malrotation results in obstruction in one instant [6]. In one case, the small intestine had multiple sites of stenosis and dilatation [29]. There are two cases of Meckel’s diverticula [2,14]. Esophageal atresia was reported in four cases [8,14,29,41]; in two of them, a distal tracheoesophageal fistula was observed [8,29]. Making the number of cases with GI malformations 29, or 44% of the total cases.

Cardiac Anomalies

Cardiac anomalies were mostly mild, starting with patent ductus arteriosus (PDA) [8,25,29,36] and patent foramen ovale [29,36]. It is worth mentioning that they were all mentioned in newborns, and only one case described the PDA as large. Other cardiac anomalies were ventricular septal defect (VSD) [2,15,20,31,45], atrial septal defect (ASD) [29,42], one of them with atrioventricular canal and tricuspid stenosis [29], teratology of Fallot [14,51], pulmonary stenosis [51], aortic coarctation [50], tricuspid insufficiency [31], and in one case, it was unspecified [6]. With a total of 18 cases and a percentage close to 28% of all reported cases.

Vascular Anomalies

In our review, we found 18 cases have been reported to have vascular anomalies, 28% of reports (Table 4), and eight of them had a single umbilical artery [8,14,29,38,39,49,56]. Dykes et al. reported in 1993 a case series study of subjects having urogenital malformations with distorted distal aortas or iliac arteries, one of whom had bladder agenesis with the aberrant aorta low in the pelvis giving rise to an aberrant left iliac artery and a left internal iliac branch absent [12]. An aberrant vascular artery connecting the right internal iliac artery to the left external iliac artery has been documented in two cases by Lowrey et al., in addition to an absent left common iliac artery in one subject [50]. Indiran et al. reported a case in which the subject had a high bifurcation of the aorta with the right external iliac artery, giving rise to what seems to be the posterior trunk of the right internal iliac artery in association with right developmental dysplasia of the hip (DDH) and gluteal muscular atrophy [35]. In a follow-up to Rodin et al.'s report, a more detailed description of the vascular anomaly was mapped, starting below the level of the superior mesenteric artery, where the abdominal aorta branches into two vessels; the smaller of the two is thought to be the distal portion of the abdominal aorta, and the larger vessel is thought to be the aberrant abdominal umbilical artery, continuing to the left as the iliac and common femoral artery. Additionally, giving a branch to the right communicates with the atretic distal aorta. It was noted that both internal iliac arteries were absent as well [57].

Vakili documented in 1973 an absent left common iliac artery and the internal and external left iliac arteries rising directly from the aorta [5]. It was reported by Kaefer and Adams that a subject had a duplicated aorta and a common vessel connecting the two internal iliac arteries [17]. Nazer et al.’s subject had a low bifurcation of the aorta, and the internal iliac arteries were absent [51]. Akdas et al. found that the subject had an absent right renal artery [11]. Lastly, an esophageal ring is formed by the left aortic arch and a posteriorly coursing aberrant right subclavian artery [46].

Muscular, Skeletal, and Neurological Anomalies

Bladder agenesis is associated with a wide array of vertebral deformities and some limb deformities. Reported vertebral, sacral, and rib malformations include, but are not limited to, butterfly vertebrae [44], block vertebrae [20,44], or hemivertebrae [45], asymmetric vertebral bodies, six lumbar vertebrae [25], additional rib [14,15,20,22], vertebral hemispondylosis [23], scoliosis [1,3,8,15,34], hemihypertrophy [15], and sacral hypoplasia or dysplasia [23,25,53], spinal bifida [1,15,16], sacral dimple [14], fused ischial bones [25], and sacral agenesis [56].

Hands and upper limb deformities: Absent thumb [14,15], accessory thumb [29], proximal insertion of the thumbs and subluxation of wrists and elbows [22], hypoplastic thumb; the other thumb showed constriction at the base with absent phalanges [29], and bilateral cubitus valgus and clinodactyly [29].

Lower limb deformities: Equinovarus deformities (clubfeet) [1,29], left foot agenesis, remnant extremity [20], rocker bottom feet [29], DDH [29,34,35,52]; one with gluteal and thigh muscle atrophy [35] and another with genu valgum [29], bilateral inguinal hernia [26], and absent fibula (hemimelia) [56].

Those patients were not reported to have neurological deficits, all but one of whom had spinal bifida and scoliosis with decreased Achilles reflexes and sensations of pain, temperature, and touch [1].

Dusmet et al. reported a case of VATER syndrome (acronym for vertebral, anal, tracheoesophageal, radial, and renal anomalies), who had a supernumerary hemivertebra with ribs and partial fusion of other ribs all on the right side; dorsolumbar scoliosis with left convexity; partial fusion of several cervical vertebrae and the sacrum; the right foot was missing two toes with syndactyly of two others (lobster claw); and a sacral caudal skin appendix, in addition to multiple anomalies affecting other systems [8]. The case of VACTERL (an acronym for vertebral deformities, anal atresia, cardiac anomalies, tracheoesophageal abnormalities, including atresia, stenosis and fistula, renal, and limbs deformities) reported by Sandal et al. had right-hand preaxial polydactyly, bilateral club foot deformity, and butterfly vertebrae [38].

Atıcı et al. had a case of mermaid syndrome (sirenomelia), with two femurs, two tibia, one fibula, one foot, and four toes; the upper limbs were without observable anomalies [49]. Another case was reported by Pandey et al. to have partial sacrococcygeal agenesis, a single lower limb, a short tibia, and absent fibula, ankle, and foot bones [39].

Out of 65 cases, 25 had muscular, skeletal, and/or neurological anomalies, or 38% of all cases.

Urinary System Anomalies

Out of the 65 cases, 61 (approximately 94%) had urinary abnormalities other than bladder and urethral agenesis. Not all cases reported had urethral agenesis.

Kidneys

Abnormalities involving the kidneys consisted mostly of hydronephrosis and absent kidneys, as well as cysts or dysplasia. There were a couple of cases of fused kidneys [12,45], and a single case of duplex kidneys [25]. A third kidney was reported in one instance [22]. At least some degree of renal impairment has been noted in 21 cases; in some cases, it was mild; in others, it led to the death of the subject. We speculate the number to be higher, as some of the subjects with more severe anomalies passed away within minutes or hours of birth, most commonly from pulmonary hypoplasia due to oligohydramnios. One reported proteinuria [15]. Three subjects were found to have hypertension; all three had a single kidney, and two had recurrent UTI; one improved after nephrostomy [4], another had refractory hypertension [26], and the third had grade II retinopathy [11].

Ureters

A few malformations of the ureter were mentioned: duplication [2,22,30] or joining at the midline [5]. Other abnormalities were noted as calcification [43], pelviureteric junction obstruction [29,48], or focal cystic dilatation [26]. Most notable was hydroureter, or tortuosity, with a total of 25 cases.

Genital Abnormalities

Males: With a total of 19 males, only two subjects had normal external genitalia for males [18,26], translating to a rate of 90% of all male reports. The malformations observed were of penoscrotal transposition in five cases [14,17,25,28,55], two of which also had a bifid scrotum: one reported descended gonads [55], the other mentioned thick vas deferens [28]. Kaefer and Adams reported one of the cases of penoscrotal transposition, in which the subject had a normal scrotum with descended testes but a small skin tag as a phallus, thus assigning a female gender despite having a karyotype of XY46 [17]. The absent phallus was noted in eight subjects, with an absent scrotum [39], an underdeveloped scrotum was noted twice [8,36], a normal scrotum [21,22,47,56], the latter reported one atrophic testis, and lastly, cryptorchidism [29] and hypospadias [13]. In one instance, the phallus was reported to be distended, with an absent scrotum and palpable testes [45]. The last case did not mention any information about genitalia [31].

Females: Ambiguous genitalia have been reported in 13 subjects [6,7,9,23,29,41,46,50,51], mostly hypertrophied clitoris or protruding skin folds. Absent labia majora and hypoplasia of the labia minora have been reported in one of the cases by Tortora et al. [6]. One case had transposition of the external genitalia: posteriorly displaced clitoris and anus with a pubic dimple [41]; similarly, another had fused labia majora with a posteriorly placed skin tag and an absent clitoris [46].

As to internal female organs, vaginal abnormalities ranged from being stenotic or with atresia [16,29,30,51,52], dilated [43], absent [20,29,50], duplicate [6], bifid [29], to blind-ended [5,10,24,37,41]. In the latter cases, they were all reported to have an absent cervix or uterus. Other cervical anomalies were of a small or stenotic cervix [1,6], or bicornuate with the vaginal duplication [6]. The uterine anomalies were hypoplasia [10,29] and bicornuate [2,3,6,9,23,51], and in three cases, one of the horns was missing [12,35,52]. Ovarian abnormalities were hypoplasia [10,29], cystic [11,24], absent unilaterally [7], and bilaterally [32]. There are 31 reported cases with internal or external genital abnormalities, representing 70% of all female cases.

There were two cases where the gender of the newborn was undetermined, as they lacked any external genitalia and neither a karyotype nor autopsy were performed [49,54].

Miscellaneous

Palmer and Russi’s patient was reported to have café au lait spots [3]. Another subject was noted to have four skin tags anterior to one of her ears and a grade II goiter, in addition to an absent kidney and multiple musculoskeletal abnormalities [15]. Ozcakir et al. reported a case of left choanal atresia in which the subject had severe oligohydramnios, leading to fatal lung hypoplasia [54]. One of Tortora’s subjects was found to have a cleft palate and strabismus [6].

Dusmet et al.’s subject had VATER; vertebral abnormalities included and were not limited to additional hemivertebrae and ribs, partial fusion, and scoliosis; anal atresia and imperforated anus; distal tracheoesophageal fistula with partial proximal esophageal atresia; tracheal cartilage anomalies; annular proximally; incomplete rings distally with partial stenosis; hypoplastic lungs; retroperitoneal ectopic adrenal tissue on microscopic examination; and wide dysplastic fontanelles. Additionally, he had dysmorphism: a broad flat nose, bilateral epicanthic folds, dysplastic low-set ears, redundant loose skin at the back of the neck, and a bilateral cleft lip with a cleft palate [8].

Dysmorphism was also noted by Khan and Walsh, with up-slanting palpebral fissures, a long philtrum, and a thin upper lip [43]. One was reported to have Treacher Collins syndrome, with epicanthic folds, posterior angulation of the ears, dolichocephalous with a prominent occiput, and a short neck [22]. Jain et al. reported a case with dysmorphic features, including a bilateral upward slant of the eyes, a depressed nasal bridge, and low-set ears. Three other cases were reported by them to have low-set ears, one of which had a webbed neck [29].

Management

The goals when treating those subjects revolved around managing the renal impairment and its complications, alleviating obstructions, genital reconstruction, urinary diversion, and achieving continence (Table 5).

**Table 5:** A summary of reports with distinct features and major surgical maneuvers, follow-up, and outcome KFT: kidney function test; UTI: urinary tract infection; F: female; M: male; mins: minutes; h: hours; d: days; w: weeks; m: months; y: years; Lt: left; Rt: right; PUJ: pelviureteric junction; VATER: vertebral, anal, tracheoesophageal, radial, and renal anomalies; VACTERL: vertebral, anal atresia, cardiac, tracheoesophageal, renal, and limbs deformities; Na: sodium; K: potassium; Cl: chloride; BUN: blood urea nitrogen; Cr: creatinine; CIC: clean intermittent catheterization; ESRD: end-stage renal disease.

Case number	Author	Article number	Gender	Age of presentation	Urinary malformations	Genital malformations	Anal anomalies	Reported syndrome or sequence	Recurrent UTI	Surgical correction	KFT	Deceased	Other comments
1	Miller	[1]	F	27 y	Hydroureteronephrosis, renal abscess	Small cervix	Poor tone		Escherichia coli, Proteus	Cutaneous ureterostomy			
2	Glenn	[2]	F	3.5 y	Hydroureteronephrosis, duplication of Lt upper collecting system, blind-ended urethra.	Bicornuate uterus				Ileal loop diversion			Improved hydronephrosis, urine collection into ileostomy bag
3	Palmer	[3]	F	23 y		Bicornuate uterus	Absent posterior fourchette		Yes	Uterostomy, afterward intraperitoneal placement of the ureters, adequate length was obtained to form a stoma	BUN 17 mg/dL, Cr 1.3 mg/dL		Persistent bacteriuria, without febrile episodes
4	Graham	[4]	F	6 w	Solitary kidney with hydronephrosis and normal urethral opening				Yes, Pseudomonas	Double barrel nephrostomy	BUN 51 mg/dL Cr 0.7 mg/dL to BUN 12.5 mg/dL and Cr 0.6 mg/dL		Failure to thrive
5	Vakili	[5]	F	10 y	Ureters join in the midline and open into the vagina, absent urethra	Blind-ended vagina, cervix absent, uterus small, ovaries normal			Escherichia coli, Proteus	Ileal conduit	BUN 19 mg/dL		
6	Tortora	[6]	F	1 m	Bilateral renal dysplasia	Prominent mons pubis, protruding skin fold, duplicate vagina and uterus			Yes	Rt nephrectomy and colonic conduit	Borderline		Follow up for 4 years: occasional UTI
7	Tortora	[6]	F	Newborn	Renal dysplasia with tortuous ureters	Absent labia majora, hypoplastic labia minora, hyperplastic skin fold in the area of the clitoris, and stenotic cervix			Yes, Escherichia coli	Ileal conduit		Passed away after candida sepsis	
8	Tortora	[6]	F	1 y	Hydroureteronephrosis					Ileal conduit			1-year follow-up: appropriate renal size according to age
9	Metoki	[7]	F	4 m	Solitary kidney with hydroureteronephrosis	Protruding skin fold, a single ovary, and a normal uterus. Single urogenital opening	Atresia				BUN 50 mg/dL, Cr 4.8 mg/dL. At 1 year old: BUN 50 mg/dL and Cr 1.6 mg/dL		
10	Dusmet	[8]	M 46 XY	Newborn	Bilateral renal dysplasia with blind ureter	Absent phallus, rudimentary, and intra-abdominal testes	Atresia	VATER				Passed after 30 minutes from birth	
11	Aragona	[9]	F	Newborn	Solitary cystic dysplastic kidney with tortuous ureter	Hypertrophied clitoris and bicornuate uterus					Progressive impairment	Passed away at 3 m with progressive renal insufficiency	
12	Krull	[10]	F 46 XX	12 d	Bilateral hydroureteronephrosis, absent urethra	Blind-ended vagina, cervix absent, uterus and ovaries hypoplastic			Yes, Klebsiella	Nephrostomies	Normal, with slight metabolic acidosis		Weight below 3rd percentile at 2 year follow up
13	Akdas	[11]	F	30 y	Solitary small kidney, absent urethra	Bilateral polycystic ovaries				Laparotomy, ureterostomy	BUN 46 mg/dL		Lost to follow up
14	Dykes	[12]	F		Fused renal ectopia	Bicornuate uterus, right side absent, ovaries and fallopian tubes present bilaterally				Laparotomy			
15	Gopal	[13]	M	2.5 y	Bilateral hydroureteronephrosis, Lt dysplastic ectopic pelvic, distended posterior urethra	Hypospadias, seminal vesicles distended bilaterally, with a cyst. The ejaculatory ducts are inserted into ureters			UTI at presentation	Left nephroureterectomy, neobladder from ileocecum	Hb 7.5 g%, urea 53.55 mmol/l, creatinine 0.25 mmol/l		Failure to thrive. Post-operation follow-up: improved KFT, gaining weight, can hold urine for ~2 hours, holding 80-100 ml
16	Cilento	[14]	M	Newborn	Solitary dysplastic kidney, urethra patent	Penoscrotal transposition					Anuric	Passed away on day 2, anuria, with normal lungs	
17	Sarica	[15]	F	12 y	Solitary ectopic hydronephrotic kidney. The urethra is blind-ended						Normal		Proteinuria
18	Bhagwat	[16]	F 46 XX	Newborn	Solitary ectopic kidney, urethra absent	Vaginal atresia	Anorectal malformation				Normal	Passed away on the 27th day due to ascending CNS infection	
19	Kaefer	[17]	M 46 XY	Newborn	Unilateral hydroureteronephrosis, the other side; cystic dysplastic. The urethra absent	Penoscrotal transposition, small skin tag anterior to the rectum, descended testis with a paucity of spermatogonia and Leydig cells					Creatinine is stable at 0.8 mg/dl		Assigned a female gender
20	Paşaoglu	[18]	M	60 y	Unilateral hydroureteronephrosis, the other side; dysplastic						Normal		Lost to follow up
21	Kasat	[19]	F	20 d	Bilateral hydroureteronephrosis, Rt enclosed by urinoma	Distal vaginal atresia, proximal part, and uterus filled with urine				Vaginostomy	Na 126 mmol/l, K 6.1 mmol/l, BUN 98 mg/dL, Cr 10 mg/dL	Passed away 5 days post-op, with massive hematuria and gastrointestinal bleeding	
22	Karaguzel	[20]	F 46 XX	Newborn	Bilateral renal ectopia with increased echogenicity, one is dysplastic	Absence of labia minora and majora and protruding skin fold in the area of the clitoris, lack of perineal openings. Absent vagina and uterus. Ovaries are normal, fallopian tube connected like a cord	Rectal atresia		UTI post-op, Escherichia coli, S. aureus	Laparotomy, sigmoid colostomy, removal of remnant extremity	BUN 74 mg/dL, Cr 1.67 mg/dL, Na 150 mmol/l, K 7.1 mmol/l, Cl 113 mmol/l	Passed away at approximately 45 days of age of sepsis and organ failure	
23	Benedetto	[21]	M 46 XY	Newborn	Bilateral renal agenesis	Absent phallus, with normal scrotum and testes	Anorectal agenesis without fistula					Passed away at 6 days from cardiac arrest	
24	Rennert	[22]	M 46 XY	Newborn	Bilateral multicystic dysplastic, third kidney also cystic dysplastic	Absent phallus with normal scrotum and testes	Posterior displacement	Treacher Collins				Passed away on day 3 from pulmonary hypoplasia	
25	Savanelli	[23]	F	4 w	Renal ectopia with tortuous ureters, "vanishing Lt kidney"	Prominent clitoris, a single perineal opening, and a bicornuate uterus	Anterior anus			Sigmoid conduit with Mitrofanoff's, vaginal reconstruction, clitoroplasty			Normal bladder capacity without reflux, at 11 years she was menstruating
26	Nazif	[24]	F	Newborn	Solitary cystic dysplastic kidney with tortuous ureter. The urethra is absent	Blind-ended vagina. Uterus and cervix absent. Fallopian tubes blind-ended, ovaries cystic			Yes	Ileocecal neobladder with Mitrofanoff's, renal transplant preemptive	Stable initially, progressed until renal failure, stable after transplant		Given somatotropin for growth. Achieved continence
27	Rodin	[25]	M 46 XY	Newborn	Echogenic solitary duplex left kidney	Penoscrotal transposition with suprapubic maldeveloped scrotum and ectopically located phallus in the sacral area					Later developed chronic kidney disease		Intestinal malrotation with bowel obstruction, on peritoneal dialysis
28	Weight	[26]	M 46 XY	Newborn	Solitary dysplastic kidney with focal cystic dilations of the ureter		Anal stenosis			Anal dilatation, hernias repair, and gastrostomy. Nephrectomy for refractory hypertension	Renal failure, hyperkalemia, Cr 3.4 mg/dL, and refractory acidosis	Deceased at 7 months	Was on peritoneal dialysis and antibiotic prophylaxis
29	Khemchandani	[27]	F	2 y	Solitary hydroureteronephrosis kidney				Yes, Escherichia coli	Ileocecal pouch with Mitrofanoff's	Creatinine 0.67 mg%		Good neobladder capacity with total continence, without reflux or urinary leak
30	Patkowski	[28]	M 46 XY	Newborn	Bilateral cystic dysplasia kidneys with urethral stenosis	Penoscrotal transposition with bifid scrotum and a thick vas deferens				Tenckhoff catheter	Progressive renal failure, Cr up to 4.8 mg/dL	Passed away at 4 m due to pneumonia	Peritoneal dialysis
31	Jain	[29]	M	Newborn	Bilateral hypoplastic dysplastic kidneys	Absent phallus with cryptorchidism	Rectal atresia	Urorectal septal malformation				Passed away at 4 h due to pulmonary hypoplasia	
32	Jain	[29]	F	Newborn		Absent perineal opening		Urorectal septal malformation				Passed away at 1 h	
33	Jain	[29]	F	Newborn	Solitary cystic dysplastic kidney with ureteric atresia and PUJ stenosis	Phallus-like structure, fused labia, a single midline perineal opening, rudimentary uterus, and ovaries	Stenotic rectal opening to cloacal sac	Urorectal septal malformation				Passed away at 10 days due to cecal perforation	
34	Jain	[29]	F	Newborn	Solitary dysplastic kidney. The urethra is absent	Vaginal agenesis and bilateral streak ovaries	Imperforated anus	Urorectal septal malformation				Passed away at 4 h due to pulmonary hypoplasia	
35	Jain	[29]	F	Newborn	Bilateral renal agenesis	Clitoral hypertrophy, no perineal opening, with bifid, atretic vagina	Imperforated anus	Urorectal septal malformation				Passed away at 3 h due to pulmonary hypoplasia	
36	Rezaie	[30]	F	1.5 y	Solitary hyperplastic hypo nephrotic kidney, with tortuous ureter. The urethra is absent	Stenotic orifice at the vaginal vestibule			Yes, Escherichia coli		Normal		Failure to thrive
37	Rezaie	[30]	F	6 y	Unilateral mild hydronephrosis, with unilateral duplicate ureter. The urethra is absent				Yes				Failure to thrive
38	Barber	[31]	M	Newborn	Unilateral cystic, bilateral dysplastic kidneys						Renal impairment	Deceased at 5 days of age, from cardiorespiratory failure and fluid overload, hypoplastic lungs	
39	Chen	[32]	F	1 m	Unilateral hypoplastic, bilateral hydronephrosis with tortuous ureters				Yes		Normal		On prophylactic antibiotics (TMP-SX)
40	Nazim	[33]	F 46 XX	8 y	Bilateral hydronephrosis, one kidney is non-functional	Urine reflux into fallopian tubes			Yes, Escherichia coli	Colonic patch (sigmoid) with Mitrofanoff's	Normal		On sodium bicarbonate. Remains continent between 3-hour intervals of CIC drainage
41	Pfister	[34]	F	3 m					Yes	Ileal pouch (20 cm), complicated by urinary leak; nephrostomy was performed and reversed later	Normal		On sodium bicarbonate, follow up at 7 years of age: thriving well, no kidney scaring or dilatation. Since the operation only one episode of UTI. Performs self-catheterization
42	Indiran	[35]	F	12 y	Solitary kidney with calyceal dilatation	Unicornuate			Yes	Awaits surgery	Normal		Poorly nourished
43	García-de León Gómez	[36]	M	Newborn	Kidneys with increased echogenicity, multi simple cystic formation, the Lt ectopic. Perineal urinary meatus	Absent phallus and scrotal raphe	Imperforated anus				Urea 26 mg/dL, creatinine 0.92 mg/dL, K 5.3 mmol/l, and Na 135 mmol/l	Passed away after cardiac and renal complications and pneumonia	
44	Baheti	[37]	F	3 y	Hydroureter	Blind-ended vagina and absent uterus				Sigmoid conduit with Mitrofanoff's	Normal		Achieved continence
45	Sandal	[38]	M 46 XY	Newborn	Bilateral renal agenesis and absent urethra	Distended penis with absent scrotum		VACTREL		Colostomy		Passed away at 36 hours due to respiratory insufficiency	Peritoneal dialysis
46	Pandey	[39]	M 46 XY	Newborn	Bilateral renal agenesis	Absent external genitalia	Imperforated anus	Sirenomelia				Passed away at 30 mins, due to respiratory distress.	
47	Priyadarshi	[40]	F 46 XX	5 y	Bilateral hydroureteronephrosis and absent urethra					Sigmoid colon and Mitrofanoff’s	Normal		Achieved continence
48	Crocoli	[41]	F 46 XX	Newborn	Hydronephrosis, small bilateral subcortical renal cysts, and a unilateral tortuous ureter	Clitoral hypertrophy and no perineal opening	Posterior displacement			Esophageal atresia repair. Planned for ileal neobladder and a Mitrofanoff's, ureteral reimplantation into the pouch, external genital resurfacing, vaginal reconstruction	Normal		
49	Pacheco-Mendoza	[42]	F	12 y	Bilateral incomplete renal duplication and unilateral ectopic kidney. The urethra is blind-ended				Yes	Ileal pouch (45 cm) with Mitrofanoff's	Cr 0.9 mg/dL to 0.8 mg/dL		On prophylactic antibiotics, follow-up reservoir showed a capacity of 300 ml
50	Ghasi	[43]	F	22 y	Bilateral hydronephrosis with unilateral calcified ureter and pelvicalyceal system, the urethra is normally positioned	Dilated vagina					Mildly elevated		Lost to follow up
51	Sailo	[44]	F	9 m	Ectopic renal lump, with absent urethra				No	Plan to perform ureter cutaneous stoma	Normal		
52	Khan	[45]	M	Newborn	Cystic dysplastic horseshoe pelvic kidney without corticomedullary differentiation, and bilateral ectopic ureters with absent urethra	Small phallus with undescended testes					Cr 2.4-3.1 mg/dL, Na 120 mmol/l, K 6.2 mmol/l	Passed away a few days later (neonate)	
53	Friedman	[46]	F 46 XX	Newborn	Unilateral cystic, bilateral dysplastic kidneys, with solitary refluxing ureter	Fused labia majora, with a skin tag posteriorly, absent clitoris (transposition of external genitalia)	Displaced anteriorly			3 y/o deceased-donor renal transplant, with a temporizing ureterostomy. At 5 y/o, she underwent the creation of an ileocecal neobladder and Mitrofanoff's, with concomitant non-refluxing implantation of her transplant ureter	ESRD, post-transplant Cr 0.6 mg/dL		Peritoneal dialysis, G-tube placement for feeding aversion, gastroesophageal reflux, chronic sinusitis, bilateral myringotomy tubes, adenoidectomy, and left-sided inguinal hernia. Mild developmental delay
54	Singh	[47]	M	Newborn	Increased renal echogenicity with multiple simple cysts and absent urethra	Absent phallus, normal scrotum, and descended testes	Anus opening absent, gluteal cleft and anal dimple not well developed	Urorectal septal malformation			Urea 60 mg/dL, Cr 1.2 mg/dL	Passed away the next day from respiratory distress	
55	Yurtcu	[48]	F	Newborn	Unilateral PUJ obstruction		Imperforated anus	Urorectal septal malformation		Colostomy, then ileocecal reservoir with pyelo-pyelostomy and Mitrofanoff's	Moderately preserved (urea 76 mg/dL, Cr 3.2 mg/dL)		
56	Atıcı	[49]	Undetermined	Newborn	Bilateral renal agenesis	Absent external genitalia	Single genital patency just below the coccyx, connected to the colon	Sirenomelia				Passed away at 8 hours of age	
57	Lowrey	[50]	F	8 y	Unilateral cystic dysplastic kidney, the other is ectopic					Bilateral nephrectomy with Charleston neobladder creation, 3 months later renal transplant	BUN 52 mg/dL, Cr 3.8 mg/dL		At presentation: 2nd percentile of weight. Six-year follow-up: achieved continence, and a functional renal transplant
58	Lowrey	[50]	F	6 y	Bilateral dysplasia kidneys	Vaginal agenesis	Imperforated anus			Studer ileal neobladder creation, bilateral open nephroureterectomies, MACE, and Monti ileovesicostomy			Peritoneal dialysis until transplant
59	Nazer	[51]	F 46 XX	1 m	Unilateral dysplastic kidney with short urethra	Distal vaginal atresia with uterus didelphys unilateral hematocolpos	Low imperforated anus and stenosis		Yes	Bilateral high anterior ureterostomies, complicated by urine leak, blockage ureter necrosis, and UTI	Slow decline, chronic kidney disease		Anemia developed later on
60	Gowtham	[52]	F	20 y	Solitary kidney with distally dilated ureter	Stenotic vaginal opening with labial adhesions			No	Vaginoplasty with adhesiolysis and laparoscopic Mainz 2 urinary pouch			Achieved continence
61	Delshad	[53]	F	3 y			Imperforated anus			Neobladder with a stoma, anorectoplasty	Normal		Achieved continence
62	Delshad	[53]	F	6 y						Neobladder (cecum with ascending colon), with stoma	Normal		Achieved continence
63	Ozcakir	[54]	Undetermined	Newborn	Bilateral renal agenesis, with the absent urethra	Smooth perineal, a small nubbin of tissue	Absent perineal orifices	Urorectal septal malformation				Deceased on the same day	Peritoneal dialysis
64	Omil-Lima	[55]	M	Newborn	Bilateral cystic dysplastic kidneys, the urethral meatus was orthotopic	Penoscrotal transposition with scrotum bifid, and descended testes bilaterally	Imperforated anus with perineal fistula			Ileal conduit	Cr 6.22 mg/dL		Peritoneal dialysis
65	Ramya	[56]	M	Newborn	Bilateral cystic dysplastic kidneys	Absent phallus with well-developed scrotum, testes one descended, the other is atrophic	Imperforated anus	Urorectal septal malformation				Passed away at 3 hours, due to respiratory failure	

Eight subjects underwent peritoneal dialysis [25,26,28,38,46,50,54,55]; only two moved on to have a renal transplant [46,50], and another had a preemptive renal transplant [24]. Three patients were placed on prophylactic antibiotics [26,32,42], and another two were given sodium bicarbonate [33,34]. In some cases, nephrectomy or ureterectomy were also performed [6,13,26,50].

Urinary diversion by ileum conduit [2,5,6,34,55], ileal Studer [50], colon conduit [6], sigmoid conduit [23], and nephrostomy, or ureterostomy, was performed in several cases [1,3,4,10,51], and in one case, a vaginostomy was performed [19].

Continence was achieved by bladder construction from a sigmoid conduit with Mitrofanoff [33,37,40], or an ileocecum neobladder with Mitrofanoff [24,27,46,48,50], and an ileal pouch with Mitrofanoff [42]. Ileocecum neobladder with anastomosis to the urethra with the use of the ileocecal valve as the neck of the bladder was performed in the case reported by Gopal [13]. One case had laparoscopic Mainz II, in which the ureters are implanted into the rectosigmoid pouch and void via the rectum [52].

Other procedures included colostomy in cases of anal atresia [20,38,48,53], followed by anorectoplasty [53], gastrostomy for feeding [26,46], vaginal reconstruction [23,52], and clitoroplasty [23].

Prognosis

At the time of reporting, 25 subjects had expired (Table 6), most of whom passed away within the first year of life. There were 14 males, nine females, and two of undetermined gender. The mortality rate was 38% in total, with 74% for males and 20% for females. The risk of mortality was factored in by kidney function, urine output, and related complications, such as oligohydramnios resulting in pulmonary hypoplasia. Nine cases reported renal impairment of some degree [9,19,20,26,28,31,36,38,42]. All six patients with bilateral renal agenesis passed away within hours of birth with respiratory insufficiency; notably, they all had complex congenital anomalies: VACTERL [38], mermaid syndrome [39,49], and urorectal septal malformation sequence [21,29,54]. The case of VATER, who had bilateral renal dysplasia, also passed away 30 minutes after birth [8]. In addition, sepsis and infections factored into the deaths of six subjects [6,16,20,26,28,36]. Bowel perforation was the cause of death in one subject [29] and cardiac arrest in another [21]. Prematurity was noted in 12 subjects as well; the youngest was 26 weeks old [49].

**Table 6:** List of mortalities with some of their main comorbidities F: female; M: male; min: minutes; d: days; w: weeks; VSD: ventricular septal defect; PDA: patent ductus arteriosus; ASD: atrial septal defect; VATER: vertebral, anal, tracheoesophageal, radial, and renal anomalies; VACTERL; vertebral, anal atresia, cardiac, tracheoesophageal, renal, and limbs deformities; Na: sodium; K: potassium; Cl: chloride; BUN: blood urea nitrogen; Cr: creatinine; KFT: kidney function test; GA: gestational age.

Case number	Author	Article number	Gender	Age of presentation	Renal anomalies	Genital malformations	Anal anomalies	Cardiac malformations	Reported syndrome or sequence	Age and cause of death	KFT	GA	Other comments
7	Tortora	[6]	F	Newborn	Renal dysplasia	Absent labia majora, hypoplastic labia minora, hyperplastic skin fold in the area of the clitoris, and stenotic cervix				Passed away after candida sepsis		37.5 w	Diabetic mother
10	Dusmet	[8]	M 46 XY	Newborn	Bilateral renal dysplasia with blind ureter	Absent phallus, rudimentary, and intra-abdominal testes	Atresia	PDA	VATER	Passed after 30 mins from birth		31 w +2	
11	Aragona	[9]	F	Newborn	Solitary cystic dysplastic kidney	Hypertrophied clitoris and bicornuate uterus				Passed away at 3 m with progressive renal insufficiency	Progressive impairment		
16	Cilento	[14]	M	Newborn	Solitary dysplastic kidney	Penoscrotal transposition		Teratology of Fallot		Passed at day 2, anuria, normal lungs	Anuric	36 w	CS due to maternal hypertension and intrauterine growth retardation. Normal amniotic fluid volume
18	Bhagwat	[16]	F 46 XX	Newborn	Solitary ectopic kidney	Vaginal atresia	Anorectal malformation			Passed away on the 27th day due to ascending CNS infection	Normal	Full term	
21	Kasat	[19]	F	20 d	Bilateral hydroureteronephrosis, Rt enclosed by urinoma	Distal vaginal atresia, proximal part, and uterus filled with urine				Passed away 5 days post-op, with massive hematuria and gastrointestinal bleeding	Na 126 mmol/l, K 6.1 mmol/l, BUN 98 mg/dl, creatinine 10.0 mg/dl, pH 7.1, HCO3 6.0 mmol/l, and calcium 7.1 mmol/l		
22	Karaguzel	[20]	F 46 XX	Newborn	Bilateral renal ectopia, with increased echogenicity, one is dysplastic	Absence of labia minora and majora and protruding skin fold in the area of the clitoris, lack of perineal openings. Absent vagina and uterus. Ovaries are normal, fallopian tube connected like a cord	Rectal atresia	Small VSD		Passed away at approximately 45 days of age of sepsis and organ failure	BUN 74 mg/dL, Creatinine 1.67 mg/dL, Na 150 mmol/l, K 7.1 mmol/l, and Cl 113 mmol/l		
23	Benedetto	[21]	M 46 XY	Newborn	Bilateral renal agenesis	Absent phallus with normal scrotum and testes	Anorectal agenesis without fistula			Passed away at 6 days from cardiac arrest		40 w	
24	Rennert	[22]	M 46 XY	Newborn	Bilateral multicystic dysplastic, third kidney also cystic dysplastic	Absent phallus with normal scrotum and testes	Posterior displacement		Treacher Collins	Passed away at day 3 from pulmonary hypoplasia		34 w	
28	Weight	[26]	M 46 XY	Newborn	Solitary dysplastic kidney		Anal stenosis			Passed at 7 months from fungal infection	Renal failure, hyperkalemia, Cr 3.4 mg/dL	36 w	Refractory hypertension, was on peritoneal dialysis
30	Patkowski	[28]	M 46 XY	Newborn	Bilateral cystic dysplasia kidneys	Penoscrotal transposition, with bifid scrotum and a thick vas deferens				Passed away at 4 months due to pneumonia	Progressive renal failure, Cr up to 4.8 mg/dL	35 w	Peritoneal dialysis
31	Jain	[29]	M	Newborn	Bilateral hypoplastic dysplastic kidneys	Absent phallus with cryptorchidism	Rectal atresia		Urorectal septal malformation	Passed away at 4 h due to pulmonary hypoplasia		34 w	
32	Jain	[29]	F	Newborn		Absent perineal opening		ASD, atrioventricular canal defect of ostium primum type, and tricuspid stenosis	Urorectal septal malformation	Passed away at 1 h with pulmonary hypoplasia and multiple congenital heart		35+5	
33	Jain	[29]	F	Newborn	Solitary cystic dysplastic kidney	Phallus-like structure, fused labia, a single midline perineal opening, rudimentary uterus, and ovaries	Stenotic rectal opening to cloacal sac		Urorectal septal malformation	Passed away at 10 days due to cecal perforation			
34	Jain	[29]	F	Newborn	Solitary dysplastic kidney	Vaginal agenesis and bilateral streak ovaries	Imperforated anus		Urorectal septal malformation	Passed away at 4 h due to pulmonary hypoplasia		39 w	
35	Jain	[29]	F	Newborn	Bilateral renal agenesis	Clitoral hypertrophy, no perineal opening, with bifid, atretic vagina	Imperforated anus		Urorectal septal malformation	Passed away at 3 h due to pulmonary hypoplasia		Full term	
38	Barber	[31]	M	Newborn	Bilateral dysplastic kidneys			VSD		Passed at 5 days of age, from cardiorespiratory failure and fluid overload, hypoplastic lungs	Renal impairment	35 w	
43	García-de León Gómez	[36]	M	Newborn	Kidneys with increased echogenicity, multi-simple cystic formation, the Lt ectopic	Absent phallus and scrotal raphe	Imperforated anus	Patent foramen ovale and PDA		Passed away after cardiac and renal complications and pneumonia	Urea 26 mg/dL and creatinine 0.92 mg/dL	38 w	Oligohydramnios, preeclampsia
45	Sandal	[38]	M 46 XY	Newborn	Bilateral renal agenesis	Distended penis with absent scrotum		ASD	VACTREL	Passed away at 36 hours due to respiratory insufficiency			Peritoneal dialysis
46	Pandey	[39]	M 46 XY	Newborn	Bilateral renal agenesis	Absent external genitalia	Imperforated anus		Sirenomelia	Passed away at 30 mins, due to respiratory distress			
52	Khan	[45]	M	Newborn	Cystic dysplastic horseshoe pelvic kidney without corticomedullary differentiation	Small phallus, with undescended testes		VSD, with tricuspid valve insufficiency		Passed away a few days later (neonate)	Cr 2.4-3.1 mg/dL, Na 120 mmol/l, K 6.2 mmol/l	35 w	
54	Singh	[47]	M	Newborn	Increased renal echogenicity, with multiple simple cysts	Absent phallus, normal scrotum, and descended testes	Anus opening absent, gluteal cleft and anal dimple not well developed		Urorectal septal malformation	Passed away the next day from respiratory distress	Urea 60 mg/dL, Cr 1.2 mg/dL	32 w	
56	Atıcı	[49]	Undetermined	Newborn	Bilateral renal agenesis	Absent external genitalia	Single genital patency just below the coccyx, connected to the colon	Aortic coarctation	Sirenomelia	Passed away at 8 hours of age with pulmonary hypoplasia and multiple congenital anomalies		26 w	
63	Ozcakir	[54]	Undetermined	Newborn	Bilateral renal agenesis	Smooth perineal, a small nubbin of tissue	Absent perineal orifices		Urorectal septal malformation	Passed on the same day with pulmonary hypoplasia and deterioration of the condition			Peritoneal dialysis
65	Ramya	[56]	M	Newborn	Bilateral cystic dysplastic kidneys	Absent phallus, with well-developed scrotum, testes one descended, the other is atrophic	Imperforated anus		Urorectal septal malformation	Passed away at 3 hours, due to respiratory failure		33 w	

The oldest patient at the time of presentation was a male at the age of 60 years, whose complaint was incontinence, for which he had fashioned a clip to be placed at his phallus. He is known to have some renal abnormalities but maintains normal kidney function. Interestingly, he has a child and has normal sexual function [18].

Females presenting at adulthood were five [1,3,11,43,52], their ability to reproduce is unknown. One was mentioned to have primary amenorrhea despite having a normal uterus but was noted to have polycystic ovaries [11]. Another was described as having a bicornuate uterus, with one of the horns having endometrioma; she underwent menarche at 13 years of age and has been having four-to-seven-week cycles since [3]. Gowtham et al. reported a case of a 20-year-old with labial adhesions, stenotic labial adhesions, and a unicornuate uterus. They reported the ovaries to be normal; it is unknown if she underwent menarche [52]. Savanelli et al. followed one subject from infancy into puberty and reported her to be menstruating normally [23].

Eight subjects have failed to thrive; all but one had UTIs. However, that subject had renal failure, a cystic-dysplastic kidney, and a corrected tetralogy of Fallot with pulmonary stenosis [50]. Another subject had a cystic-dysplastic single kidney; she was given somatotropin, which aided her growth until she received a preemptive renal transplant, allowing her to continue to thrive [24]. The remainder cases all had hydronephrosis [4,10,13,30,35]. In one case, a subject had mild developmental delay; she was diagnosed at birth and received a renal transplant at three years of age [46].

Discussion

Embryology

The bladder formation starts embryologically between the 4th and 7th weeks; the cloaca is divided by the urorectal spectrum into the rectum posteriorly and the urogenital sinus anteriorly; the latter divides into three segments [58]: the vesical part gives most of the bladder and is continuous with the allantois; the pelvic part gives the whole urethra in females and the prostatic part of the urethra in males; the phallic part gives the penis or the clitoris.

The allantois arises as a diverticulum of the yolk sac and is responsible for waste elimination and gas exchange. It also contributes to the formation of the umbilical cord, umbilical vessels, and placenta as it regresses between the 6th and 8th weeks of gestation, and the remnant is located between the two arteries within the cord. The intra-abdominal segment will constrict into the urachus, a thick, fibrous cord, and later obliterate to become the median umbilical ligament [59].

During embryonic development, initially the umbilical arteries branch ventrally from the dorsal aorta to the placenta in close association with the allantois. During the fourth week, a secondary connection forms from the common iliac arteries to the umbilical arteries, and it loses the primary dorsal aortic. After birth, the distal part of the umbilical artery obliterates, leaving the medial umbilical ligament, and the proximal part becomes the internal iliac arteries and superior vesical arteries [60]. Interestingly, the developing kidney initially receives its blood supply from the common iliac arteries and later from the distal aorta [58].

Blood Supply

The blood supply of the bladder comes from the superior, middle, and inferior vesical arteries. The anterior division of the internal iliac artery gives rise to multiple arteries; the first is the umbilical artery, which gives rise to the superior vesical artery and the middle vesical, the latter may branch from the superior vesical [61]. The inferior vesical artery is a direct branch of the anterior division of the internal iliac artery; it may share a trunk with the middle rectal artery. In addition to supplying the inferior part of the bladder, the inferior vesical artery supplies the prostate, seminal vesicles, and sometimes the ductus deferens [62]. On occasion, it might be a branch of the internal pudendal artery [63], and it is usually observed more commonly in males [64]. The bladder may receive additional blood supply from the obturator artery and the inferior gluteal artery [62]. The veins from the urinary bladder drain into the internal iliac vein [61].

The internal iliac artery has visceral and parietal branches; the visceral branches supply the urinary bladder, rectum, and urethra; in males, they additionally supply the prostate, ductus deferens, seminal vesicles, and ejaculatory ducts; and in females, they supply the uterus and vagina. The parietal branches supply musculoskeletal structures in the thigh, hip joint, and gluteal region [62].

Etiology

The exact pathogenesis of bladder agenesis is still unknown, and it is yet to be determined whether bladder agenesis and its associated malformations are due to a common etiology with high variability or whether different factors can lead to bladder agenesis.

Dykes et al. suggested that vascular abnormalities and bladder agenesis have the same underlying etiology [12], while Lowrey et al. propose that the organ's maldevelopment is provoked by vascular variations [50]. Research on the etiology of bladder agenesis is scarce. However, researchers have noticed overlapping with some syndromes or sequences involving caudal dysgenesis, and some of the reports we have reviewed were of bladder agenesis in association with the urorectal septal malformation sequence, VATER/VACTERL, sirenomelia, and Treacher Collins syndrome.

One of the theories dealing with caudal dysgenesis generally and sirenomelia specifically is vitelline vascular steal; vitelline vessels are branches of the dorsal aorta in the developing fetus and form a vascular network over the yolk sac. When a coalescence single large vitelline artery arising from the aorta assumes the function of the umbilical arteries and forms a single umbilical artery, the blood flow would be diverted to the placenta rather than the caudal portion of the embryo, leading to nutritional deficits and structural malformations [65]. A severe type would result in lower limb amelia and the absence of the lower abdominal and pelvic structures, and a lesser form might lead to a wide variety of genitourinary and gastrointestinal defects [66].

A single umbilical artery (SUA) can be isolated when the normally derived umbilical arteries fuse prior to exiting the umbilical ring or when one of them gets atrophied [66]. Depending on the number and types of vessels in the umbilical cord, SUA can be classified into four types [67]: type I: one allantoic umbilical artery (right or left), with the left umbilical vein (two vessels). Associated with central nervous system or genitourinary malformations, a short umbilical cord, and acarida. Type II: one vitellinic umbilical artery from the superior mesenteric artery, with the left umbilical vein (two vessels). Associated with caudal regression, sirenomelia, and anal agenesis. Type III: one allantoic or vitellinic umbilical artery, with the left umbilical vein and the right anomalous umbilical veins (three vessels). Associated with fetal anomalies such as renal agenesis, unicornuate uterus, hydranencephaly, and ipsilateral limb reduction. Type IV: one allantoic or vitellinic umbilical artery and right anomalous umbilical veins (two vessels). Associated with spontaneous miscarriage.

A single large umbilical cord artery was noted in a third of cases with VACTERL (vertebral, anal, cardiac, trachea-esophageal, renal, and limbs) association, urorectal septal malformation (URSM) sequence, OEIS (omphalocele, exstrophy of the cloaca, imperforate anus, and spinal defects) complex, half of the cases with limb body wall defect (LBWD), and in almost all the cases of sirenomelia (mermaid syndrome). In some of the few reports that had two umbilical arteries, the vessels had disproportionate sizes or one was occluded. Those entities might be a continuum of malformations in the caudal structures due to environmental or genetic factors preventing normal urorectal septum and allantois development [66]. In extremely rare cases, two caudal defects may occur in the same fetus. In a case reported by Kitova et al., a fetopathological dissection of a fetus revealed mermaid syndrome with VACTERL-H syndrome: (V) myelomeningocele; (A) anal atresia; (C) cardiac defects: absent; (TE) tracheoesophageal fistula; (R) single umbilical artery; bilateral renal and ureteric agenesis; bladder agenesis; agenesis of external genitals; agenesis of the female internal gonad; (L) monkey fold of the left palm; (H) hydrocephalus [68]. As rare as bladder agenesis is, it happens to be a common finding in the extremely rare cases of sirenomelia [66].

To consider vitelline vascular steal as the pathogenic cause of the caudal malformations, certain criteria must be met: (1) the steal artery arises above the bifurcation of the abdominal aorta; (2) the coalescence artery dominates in size over other arteries below its origin; and (3) it is present in the umbilical cord as the only or dominant artery. It is not clear yet whether vascular steal is an independent phenomenon or whether some predisposing factors can be linked as well, such as environmental factors or as a response to genetic damage of the allantois or caudal structures, and the vitelline vascular is a rescue response [66].

The lack of reports of vascular abnormalities in some reports we have reviewed might not mean the absence of actual anomalies; a lot of the cases were discovered beyond the neonatal period, and the status of the umbilical cord could not be determined, in addition to the number of patients being lost for follow-up or the family refusing autopsy. From the details in the reports, it is hard to determine if they had vitelline vascular steal without knowing the size and dominance of those vascular anomalies and their presence in the umbilical cord in a retrospective manner.

Some of the cases we have reviewed were reported to have the URSM sequence, which includes absent anal and perineal openings in association with colonic, urogenital, and lumbosacral malformations and ambiguous genitalia. It can be complete, lacking any openings, or partial, where the common cloaca is drained by a single opening with anal atresia, or urogenital, with an anus and single urogenital orifice, which is seen only in females, or lastly, an anteriorly placed anus with hypoplastic perineum [69]. It is thought to be due to the incomplete subdivision of the cloaca and/or the lack of cloacal membrane breakdown [70].

From our review, we believe that possibly around 20 of the reports might have had URSM of all four subtypes but have not been identified as such (Table 5), and two fulfill the minimum diagnostic criteria of VACTERL associations, in addition to three cases where VACTERL and URSM have overlapped, and the subjects were identified with only one.

Nine cases were reported as URSM, two with sirenomelia, one VATER, and another VACTERL, in total, making up 20% of all cases. If we take into consideration the ones we have identified, it will bring the percentage to 54%.

Other pathogenic mechanisms have been raised as possible etiologies of caudal structure malformations or sequences: deficiency of the embryonic disc, deficiency of the caudal mesoderm, early amnion rupture and amniotic bands, intrauterine constraint, gene mutations, and genomic imbalance [66].

In one instance, bladder agenesis was associated with intrauterine Zika virus infection at 16 weeks of gestation, along with hypoplastic kidneys, anhydramnios, and intrauterine growth restriction [71]. Calin et al. reported a case of monochorionic-diamniotic twins; the first terminated at 23 weeks of gestation with an absent bladder, urethra, vagina, anorectal atresia, omphalocele with amniotic band over limbs, and the umbilical cord; the cord was short and thin, and the second was mummified with normal internal organs [72].

Liu et al.'s studies on the effect of Adriamycin on rats resulted in bladder agenesis in 100% of rats exposed during gestational day six, compared to 83% and 77% when exposed at days seven and nine, respectively [73], stating that it was primary agenesis rather than secondary resorption of the bladder [74], and not only bladder agenesis was observed in those exposed rats but a whole spectrum of cloacal and urogenital anomalies [75].

With over half of all cases falling under the umbrella of caudal dysgenesis syndromes, one cannot ignore the fact that the other half of the cases are not yet identified as part of any syndrome or sequence, and in one case, it was isolated [53], raising the question whether there are multiple etiologies for bladder agenesis or one underlying cause resulting in highly variable anomalies.

Study limitations

While this systematic review contributes valuable insights, it is important to recognize its inherent limitations. The incorporation of case reports, while informative, inherently limits the ability to extrapolate overarching conclusions due to their retrospective and non-randomized nature. This introduces the potential for overinterpretation and biases. Furthermore, the compilation of data from diverse sources introduces a lack of standardization. Disparities become evident across crucial aspects, diagnostic procedures, and follow-up protocols. As a result, the overall robustness of the findings is moderated.

It is worth noting that these limitations provide opportunities for future research. To address the challenge of standardization, future studies could prioritize the establishment of uniform diagnostic criteria and follow-up procedures. Additionally, efforts to minimize bias could involve rigorous reporting guidelines and a more structured approach to case presentation.

Perhaps starting with identifying a common denominator will lead to an underlying cause, answering that question and aiding in mapping out the sequence. In addition to identifying its predisposing factor, this needs to be accomplished for the field to move toward a more comprehensive understanding of the rare anomaly.

## Conclusions

Bladder agenesis is found to be associated with a wide spectrum of malformations, including urinary system malformations in 93% of the cases, most commonly hydronephrosis, absent, cystic, or dysplastic kidneys; and genital malformations or ambiguous genitalia in 90% of male subjects, most notably an absent phallus and penoscrotal transposition. As to the females, 70% were reported with internal or external reproduction organ abnormalities, such as ambiguous genitalia, vaginal stenosis or blind-ended, and ureteral bicornuate or agenesis, for a total of 77% of all cases; gastrointestinal anomalies in 44%, most notable being imperforated anus; musculoskeletal malformations in 38%; and cardiac malformations in 28% of the cases; similarly, 28% of all subjects had vascular abnormalities.

Only one of the reviewed cases was isolated, and on the other side of the spectrum were the ones with complex congenital anomalies who passed away within minutes of birth with pulmonary hypoplasia, the most common cause of death. Of course, the spectrum extends beyond that to reach infants who do not make it to birth. Notably, bladder agenesis was found as part of different caudal dysgenesis syndromes, such as sirenomelia, VATER/VACTERL, and urorectal septum malformation sequences. Due to the complexity of the associated congenital anomalies, bladder agenesis has a mortality rate of 38%, mostly during the first year. It has been attributed that the difference between the live births between females and males, with a ratio of 2.3:1, is due to the complexities of the associated congenital anomalies, which is reflected by the discrepancy in the mortality rates, with 74% for males and 20% for females.
